# Supplementary material for: Effects of microsurface structure of bioactive nanoparticles on dentinal tubules as a dentin desensitizer
Source: PLoS One. 2020 Aug 19;15(8):e0237726. doi: 10.1371/journal.pone.0237726 (PMC7437925; doi:10.1371/journal.pone.0237726)
Supplement: S3 File — (PDF) [file pone.0237726.s003.pdf]

CERTIFICATE OF TRANSLATION

June 28, 2020

This document certifies that the paper titled

Effects of microsurface structure of bioactive nanoparticles on dentinal tubules as a dentin desensitizer

has been translated and edited for language by professionals at Editage, a division of Cactus Communications, under the job code DOSVP_1.

Disclaimer: We do not bear responsibility for revisions made to the document after our translation on **February 7, 2020.**

Vikas Narang

Vikas Narang,
Chief Operating Officer,
Editage

INFORMATION SECURITY MANAGEMENT SYSTEM
DNV·GL
ISO/IEC 27001

Worldwide	Japan	Korea	China	Brazil	Taiwan
request@editage.com	submissions@editage.com	submit-korea@editage.com	fabiao@editage.cn	inquiry-brazil@editage.com	submitjobs@editage.com
+1 877-334-8243	+81 03-6868-3348	1544-9241 (free dial)	400-005-6055	0880-892-20-97	02-2657-0306
www.editage.com	www.editage.jp	www.editage.co.kr	www.editage.cn	www.editage.com.br	www.editage.com.tw
