# Supplementary material for: Effects of microsurface structure of bioactive nanoparticles on dentinal tubules as a dentin desensitizer
Source: PLoS One. 2020 Aug 19;15(8):e0237726. doi: 10.1371/journal.pone.0237726 (PMC7437925; doi:10.1371/journal.pone.0237726)
Supplement: Attachment — Submitted filename: Response to reviewers.docx [file pone.0237726.s004.docx]

PONE-D-20-07389
Effects of microsurface structure of bioactive nanoparticles on dentinal tubules as a dentin desensitizer
PLOS ONE

***Dear editor in- chief Hamid Reza Bakhsheshi-Rad***

Thanks for a thorough reading and constructive criticism of our manuscript and allowing the opportunity to revise and resubmit. We are pleased to submit the revised article. On the following pages, we responded to comments from editors and reviewers.

Dear Dr. Son,

Thank you for submitting your manuscript to PLOS ONE. After careful consideration, we feel that it has merit but does not fully meet PLOS ONE’s publication criteria as it currently stands. Therefore, we invite you to submit a revised version of the manuscript that addresses the points raised during the review process.

It has been reviewed by experts in the field and we request that you make Moderate Revisions before it is processed further. Please find your manuscript and the review reports at the following link:

Reviewer & Editor Comments
This contribution would require moderate revision before it could be reconsidered for publication in PONE. Some of my comments and questions on this manuscript are as follows:
Q1: Section including “Introduction”; “Materials and methods”; “Results”; “Discussion” and corresponding subsection is required numbering.

A1(Answer1): We added numbering to each section and corresponding subsection.

Q2: Some of subsections should be merged together.

A2: We have merged some subsections. Please check the revised file.

In “materials and methods” section, “Synthesis of dense bioactive glass nanoparticles” and “Synthesis of mesoporous bioactive glass nanoparticles” were combined to “Synthesis of dense bioactive glass nanoparticles and mesoporous bioactive glass nanoparticles”. In “Results” section, “XRD” and “FTIR” were combined to “XRD and FTIR”.

Q3: “FESEM evaluation of dentinal tubule occlusion” section should be shifted to the “Materials characterizations” section.

A3: We thought that applying the bioactive glass on the dentinal tubules to evaluate the dentinal tubules' sealing capacity was part of the clinical application of bioactive materials. Therefore, instead of shifting to the 'materials characterization' section, it would be better to place it as a separate subtitle to highlight its clinical relevance.

Q4: The authors should have a consistent font size throughout the manuscript; surprisingly some part of the main text is in a BOLD type.

A4: We checked the whole manuscript thoroughly and corrected the wrong text type.

Q5: The author stated that “MBGNs with a large surface area have more exposed Si-OH, which leads to greater hydroxyapatite deposition [25, 26, 27]”. In this regard, the author should further highlighted the role of Si and OH ions on the acceleration of apatite deposition when MBGNs exposed to the physiological solution

A5: Thanks for pointing out. We added additional information regarding the role of Si and OH ions on the acceleration of apatite deposition when MBGN is exposed to physiological solutions. The added contents are as follows. : 1)The first silicate ion of the Si-OH group was combined with the calcium ion in the SBF solution. 2)In addition, calcium ions of calcium silicate combine with phosphate ions of SBF to form amorphous calcium phosphate. 3)This amorphous calcium phosphate was transformed into crystalline apatite. 4)MBGN with a large surface area had more exposed Si-OH and this feature accelerated the deposition of hydroxyapatite. Check the revised manuscript.

Q6: I strongly recommended the author present the a schematic image regarding the preparation, and characterization of bioactive glass nanoparticle prepared by sol-gel method (Fig. 1).

A6: We added schematic images of the fabrication and characterization of bioactive glass nanoparticles and schematic images of material methods (Fig. 1).

Q7: The quality of the FTIR spectra of MBGN and dense BGN (Fig. 3) should be improved.

A7: We replotted most figures, including FTIR spectra of MBGN and dense BGN. (Fig. 4)

Q8: Fig. 6, Fig. 7 and Fig. 8 should be merged together and presented as Fig. 6a, b and c. The Y and X axis of all figures beside their quality should be significantly improved. ALL figures should be replotted.

A8: We agree that Figures 6, 7, and 8 should be combined. We combined Figures 6, 7, and 8 into Figure 6.

Q9: The quality of Fig. 14 should be improved.

A9: We replotted Fig. 14 to improve the quality of the graph.

Q10: Surprisingly small reference to PLOS ONE in the literature despite the large relevant literature there. This should be improved. There are several important papers in the recent literature of PLOS ONE.
“[6,7,8,9]” should replace with “[6-9]”
“[25, 26, 27]” should replace with “[25-27]”
A10: We could find the recent literature of PLOS ONE about bioactive glasses and added two more references published in PLOS ONE(reference 9,21). Also, we have reformatted the reference part in the manuscript according to the above style guidelines.

Please submit your revised manuscript by Jul 26 2020 11:59PM. If you will need more time than this to complete your revisions, please reply to this message or contact the journal office at plosone@plos.org. When you're ready to submit your revision, log on to https://www.editorialmanager.com/pone/ and select the 'Submissions Needing Revision' folder to locate your manuscript file.

Please include the following items when submitting your revised manuscript:

- A rebuttal letter that responds to each point raised by the academic editor and reviewer(s). You should upload this letter as a separate file labeled 'Response to Reviewers'.
- A marked-up copy of your manuscript that highlights changes made to the original version. You should upload this as a separate file labeled 'Revised Manuscript with Track Changes'.
- An unmarked version of your revised paper without tracked changes. You should upload this as a separate file labeled 'Manuscript'.

If you would like to make changes to your financial disclosure, please include your updated statement in your cover letter. Guidelines for resubmitting your figure files are available below the reviewer comments at the end of this letter.

If applicable, we recommend that you deposit your laboratory protocols in protocols.io to enhance the reproducibility of your results. Protocols.io assigns your protocol its own identifier (DOI) so that it can be cited independently in the future. For instructions see: <http://journals.plos.org/plosone/s/submission-guidelines#loc-laboratory-protocols>

We look forward to receiving your revised manuscript.

Kind regards,

Hamid Reza Bakhsheshi-Rad
Academic Editor
PLOS ONE

Journal Requirements:

When submitting your revision, we need you to address these additional requirements.

1. Please ensure that your manuscript meets PLOS ONE's style requirements, including those for file naming. The PLOS ONE style templates can be found at

https://journals.plos.org/plosone/s/file?id=wjVg/PLOSOne_formatting_sample_main_body.pdf and

https://journals.plos.org/plosone/s/file?id=ba62/PLOSOne_formatting_sample_title_authors_affiliations.pdf

A1: We have reformatted the manuscript according to the above style guidelines.

2. Thank you for providing information about the ethics approval and informed patient consent.

However, we additionally ask that you update your Methods section to specify what type of consent was given (i.e., written, verbal, etc.)

We also ask you to provide the number of the IRB-approved protocol in both the Methods and the Ethics statement, if available.

A2: Thank you for your concern about ethical approval and your consent for the study. Our study was a deliberation exemption study. The deliberation exemption criteria included: Researchers do not know the personally identifiable information of human-derived donors, and the results obtained through the study are not related to the donor's genetic characteristics. Therefore, we conducted a study based on donor consent exemption. We revised the information regarding the plaintiff's ethical approval and prepared and posted an ethical statement file.

3. Please ensure that you refer to Figure 1 in your text as, if accepted, production will need this reference to link the reader to the figure.
A3: We agree to offer link of the figure to the reader. Let us know if we need to revise more.

[Note: HTML markup is below. Please do not edit.]

Reviewers' comments:

Reviewer's Responses to Questions

**Comments to the Author**

1. Is the manuscript technically sound, and do the data support the conclusions?

The manuscript must describe a technically sound piece of scientific research with data that supports the conclusions. Experiments must have been conducted rigorously, with appropriate controls, replication, and sample sizes. The conclusions must be drawn appropriately based on the data presented.

Reviewer #1: Yes

2. Has the statistical analysis been performed appropriately and rigorously?

Reviewer #1: Yes

3. Have the authors made all data underlying the findings in their manuscript fully available?

The [PLOS Data policy](http://www.plosone.org/static/policies.action#sharing) requires authors to make all data underlying the findings described in their manuscript fully available without restriction, with rare exception (please refer to the Data Availability Statement in the manuscript PDF file). The data should be provided as part of the manuscript or its supporting information, or deposited to a public repository. For example, in addition to summary statistics, the data points behind means, medians and variance measures should be available. If there are restrictions on publicly sharing data—e.g. participant privacy or use of data from a third party—those must be specified.

Reviewer #1: Yes

4. Is the manuscript presented in an intelligible fashion and written in standard English?

PLOS ONE does not copyedit accepted manuscripts, so the language in submitted articles must be clear, correct, and unambiguous. Any typographical or grammatical errors should be corrected at revision, so please note any specific errors here.

Reviewer #1: No

5. Review Comments to the Author

Please use the space provided to explain your answers to the questions above. You may also include additional comments for the author, including concerns about dual publication, research ethics, or publication ethics. (Please upload your review as an attachment if it exceeds 20,000 characters)

Reviewer #1: The manuscript entitled “Effects of microsurface structure of bioactive nanoparticles on dentinal tubules as a dentin desensitizer” by Yang-Jung Choi et al is a nice submission on DH. The manuscript is acceptable with some minor changes
1. Combine line 2 and 3.

We have combined the line 2 and 3 in the first page.

2. Abstract is too long and contain unnecessary information.

We agree that the Abstract is too long and contains unnecessary information. We have removed unnecessary experimental details and shortened the length of the Abstract.

3. Experimental detail about the individual component cytotoxicity should be given.

Thank you for the reviewer's request for experimental details of individual component cytotoxicity. In our experiments, we investigated cell viability after direct exposure to bioactive glasses according to ISO 10993-5. We have not investigated the cytotoxicity of individual components of bioactive glasses, which may be a limitation of the study. To clarify the individual component cytotoxicity of bioactive glasses, we believe more research is needed. We have added comments on the limitations of our manuscript.

4. Figure 1 need to be reconstructed in a visual experimental form rather than like a flow chart.

We have changed the type of figure from the flow chart to an experimental visual form. Please check the figure 2.

5. Figure 2A, Provide detail of peak in the picture.

We acknowledge that usually, XRD graph includes detail of peak to specify the crystal structure of materials. Since our specimen (bioactive glasses) had an amorphous structure, we concluded that it was not meant to provide detail of peak in the picture. We attached the raw data of the materials (S1, S2 file). Please check.

6. Figure 6,7, 8 need to be made more stylish to look good for the readers.

We agree that the Fig. 6, 7, 8 need to be made more stylish. Therefore, we replotted most figures. We combined Fig. 6, 7, 8 express the result of ion dissolution in the one figure (Fig 6.)

7. Other than these minor flaws, author should also correct the English mistakes which are often present in the manuscript.

We have checked the manuscript again. Also, we added a translation certificate(S3 file).

8. Recent literatures need to be cited. Some suggested one are: DOI: 10.1021/acs.chemrestox.8b00129, DOI: 10.1039/c8tx00112j

  We have added a new reference at the discussion part.

6. PLOS authors have the option to publish the peer review history of their article ([what does this mean?](https://journals.plos.org/plosone/s/editorial-and-peer-review-process#loc-peer-review-history)). If published, this will include your full peer review and any attached files.


If you choose “no”, your identity will remain anonymous but your review may still be made public.


**Do you want your identity to be public for this peer review?** For information about this choice, including consent withdrawal, please see our [Privacy Policy](https://www.plos.org/privacy-policy).

Reviewer #1: No


[NOTE: If reviewer comments were submitted as an attachment file, they will be attached to this email and accessible via the submission site. Please log into your account, locate the manuscript record, and check for the action link "View Attachments". If this link does not appear, there are no attachment files.]

While revising your submission, please upload your figure files to the Preflight Analysis and Conversion Engine (PACE) digital diagnostic tool, <https://pacev2.apexcovantage.com/>. PACE helps ensure that figures meet PLOS requirements. To use PACE, you must first register as a user. Registration is free. Then, login and navigate to the UPLOAD tab, where you will find detailed instructions on how to use the tool. If you encounter any issues or have any questions when using PACE, please email PLOS at [figures@plos.org](mailto:figures@plos.org). Please note that Supporting Information files do not need this step.
